# Development and Evaluation of Ethosomes Loaded with *Zingiber zerumbet* Linn Rhizome Extract for Antifungal Skin Infection in Deep Layer Skin

**DOI:** 10.3390/pharmaceutics14122765

**Published:** 2022-12-09

**Authors:** Kampanart Huanbutta, Napapat Rattanachitthawat, Kunlathida Luangpraditkun, Pornsak Sriamornsak, Vivek Puri, Inderbir Singh, Tanikan Sangnim

**Affiliations:** 1 School of Pharmacy, Eastern Asia University, Thanyaburi 12110, Thailand; 2 Faculty of Pharmaceutical Sciences, Burapha University, Chonburi 20131, Thailand; 3 Department of Pharmaceutical Technology, Faculty of Pharmacy, Silpakorn University, Nakhon Pathom 73000, Thailand; 4 School of Pharmacy, Chitkara University, Baddi 174103, India; 5 Chitkara College of Pharmacy, Chitkara University, Rajpura 140401, India

**Keywords:** ethosome, *Zingiber zerumbet* Linn, antifungal, skin permeation, fungal infection

## Abstract

Skin fungal infection is still a serious public health problem due to the high number of cases. Even though medicines are available for this disease, drug resistance among patients has increased. Moreover, access to medicine is restricted in some areas. One of the therapeutic options is herbal medicine. This study aims to develop an ethosome formulation loaded with *Zingiber zerumbet* (L.) Smith. rhizome extract for enhanced antifungal activity in deep layer skin, which is difficult to cure. Ethosomes were successfully prepared by the cold method, and the optimized formulation was composed of 1% (*w*/*v*) phosphatidylcholine and 40% (*v*/*v*) ethanol. Transmission electron microscope (TEM) images revealed that the ethosomes had a vesicle shape with a diameter of 205.6–368.5 nm. The entrapment of ethosomes was 31.58% and could inhibit the growth of *Candida albicans* at a concentration of 312.5 μg/mL. Finally, the ethosome system significantly enhanced the skin penetration and retention of the active compound (zerumbone) compared with the liquid extract. This study showed that *Z. zerumbet* (L.) rhizome extract could be loaded into ethosomes. The findings could be carried over to the next step for clinical application by conducting further in vivo penetration and permeation tests.

## 1. Introduction

Nearly a billion people worldwide suffer from skin, nail, and hair fungal infections. The most common fungal diseases are fungal nail infections, ringworm, vaginal candidiasis, and *Candida* infections of the gastrointestinal tract [1]. *Candida, Cryptococcus*, and *Aspergillus* are the most prevalent organisms responsible for life-threatening fungal infections in humans [2]. Despite the accessibility of drugs for fungal infection, the increase in morbidity and mortality associated with invasive fungal infections is due to the increasing number of antifungals with a small safety margin. Furthermore, certain fungi are growing resistant to treatment, making the infections difficult to treat [3]. Hence, the development of a new antifungal agent is critical to provide a treatment option for this disease.

Skin fungal infections can be classified as either superficial or deep. Superficial fungal infections, including dermatophytes, have an affinity for keratin and therefore are typically limited to either the epidermis or adnexal structures. Deep fungal infections affect deep structures, including internal organs, and continue to be an important cause of morbidity and mortality, especially in transplant recipients and other immunosuppressed patients. These diseases are extremely difficult to cure because the antifungal drugs must penetrate the stratum corneum, which is challenging due to the limited solubility of the drugs used to treat this disease and the difficulty in penetrating the skin [4].

Antifungal drug resistance and deep skin fungal infections can be remedied by discovering natural products or extracts and distributing them with an appropriate drug delivery system. Among various herbal plants, *Zingiber zerumbet* (L.) Smith. exhibits antifungal, antioxidant, and anti-inflammatory properties, all of which are essential for treating skin fungal infections [5]. *Z. zerumbet* (L.) is a ginger species with leafy stems and reaches a height of around 1.2 m. [6]. Although it originated in Asia, it is now found in a variety of tropical nations. Our previous study found that the minimum bactericidal and fungicidal concentrations of hexane and dichloromethane extracts against *Staphylococcus epidermidis* and *Candida albicans* were 31.25 and 62.5 µg/mL, respectively [5]. Moreover, the ethanol extract outperformed the dichloromethane and hexane extracts in terms of antioxidant activity as measured by ABTS and DPPH assays and showed anti-inflammatory properties as determined by protein denaturation test.

Even though the activity of *Z*. *zerumbet* (L.) rhizome is considerable, the extremely low solubility of its extract limits its application. Therefore, advanced topical formulations that significantly enhance skin penetration are required to improve the therapeutic efficacy of the extract. Ethosome is a soft and malleable vesicle primarily composed of phospholipids, ethanol, and water. The increased flexibility of vesicular membranes due to the addition of ethanol enables the elastic vesicles to squeeze through pores with diameters smaller than their own. Ethosomal systems are significantly superior to conventional liposomes and hydroalcoholic solutions for the delivery of substances to the skin in terms of quantity and depth [7,8,9].

This study aims to develop and evaluate ethosomes containing *Z*. *zerumbet* (L.) rhizome extract for the treatment of antifungal skin infections in deep layer skin. The rhizome of *Z*. *zerumbet* (L.) was extracted with hexane, and ethosomes containing this extract were prepared using the cold method. The ethosomes’ size, size distribution, zeta potential, and morphology were investigated, and the percentage of extract loading and antifungal activities of the prepared ethosomes were analyzed. Finally, the efficiency of ethosome penetration in pig skin was evaluated.

## 2. Materials and Methods

### 2.1. Materials

Ethanol (batch no. 20060068) and hexane (batch no. 21070007) were purchased from RCI Labscan, Thailand. Phosphatidylcholine from soya lecithin (batch no. MM3D32) and polyethylene glycol 4000 (PEG 4000) (batch no. J7F3RW) were acquired from MySkinRecipes, Thailand. Zerumbone (batch no. S00321110, purity ≥ 98%) were obtained from ChemFaces (Wuhan, China). All other chemicals and reagents were of analytical grade.

### 2.2. Methods

#### 2.2.1. *Z. zerumbet* (L.) Extract

The *Z*. *zerumbet* (L.) rhizomes were identified by Dr. Boonyadist Vongsak, Faculty of Pharmaceutical Sciences, Burapha University, Thailand. The voucher specimens (KM No. 0415001, KM No. 0415002, and KM No. 0415003) were deposited at the Faculty of Pharmaceutical Sciences, Burapha University, Thailand.

Fresh rhizomes of *Z*. *zerumbet* (L.) were collected in Chantaburi Province last February 2022 and then cleaned with water. The rhizomes were milled and then dried at 50 °C for 48 h. For extraction, *Z*. *zerumbet* (L.) rhizomes were soaked with hexane for 24 h. The extract was then filtered, evaporated using a rotary vacuum evaporator (R-100, Buchi, Taito, Japan), and stored at 2 °C–8 °C for further ethosome preparation.

#### 2.2.2. Ethosome Preparation

Ethosomes were prepared using the cold method. Soya lecithin, PEG 4000, and *Z. zerumbet* (L.) rhizome extract were dissolved in ethanol, and the volume of the prepared ethosome was adjusted by adding purified water. The mixture was then stirred at 30 °C to allow ethosome formation. The obtained ethosomes were sonicated for 15 min. To prevent structural damage to the ethosome, the applied sonication duration was 5 min per round for 15 min of sonication (Ultrasonic Sonicator, GT-SONIC-20, PASTEL, Shenzhen, China). Variable amounts of soya lecithin, PEG 4000, and ethanol were used, as shown in Table 1, and the effect of sonication time was also evaluated. The optimized blank ethosome formulation was selected for the loading of *Z. zerumbet* (L.) rhizome extract at a concentration range of 2.5–125 mg/20 mL.

#### 2.2.3. Physical Evaluation of Ethosomes

- Vesicle size, size distribution, and zeta potential

The vesicle size, size distribution, and zeta potential of blank and extract-loaded ethosomes were monitored by Zetasizer (MAL1070387, Malvern, UK). The samples were diluted to 0.5% *w*/*v* using deionized water as a solvent and then agitated for 3 min before measurement. The average and standard deviation of the measurements for three batches of samples were reported.

- Morphology

The surface morphology was determined by transmission electron microscopy (TEM; Tecnai 20, Philips, Eindhoven, The Netherlands). For TEM examination, a drop of the sample was placed on a carbon-coated copper grid and then negatively stained with 1% aqueous solution of phosphotungstic acid after 15 min. The grid was air dried thoroughly, and the samples were viewed on a TEM.

#### 2.2.4. Entrapment Efficiency (EE) of Ethosomes

Zerumbone, which is the major constituent of *Z. zerumbet* (L.) rhizome extract was used as an active substance marker to indirectly determine the EE of the ethosome system. First, the free zerumbone was separated from the ethosome dispersion by 90 min of centrifugation (Centrifuge, MPW-260R, MPW MED Instruments, Warsaw, Poland) at 15,000 rpm and 4 °C. After that, 900 µL of ethanol were added to 100 µL of the supernatant containing unentrapped zerumbone. Then, the unentrapped zerumbone in the sample was examined by high-performance liquid chromatography (HPLC; SPD-M20A, Shimadzu, Nakagyo, Japan) and quantified using a slightly modified, previously validated method with the limit of quantification (LoQ) of 2.5 μg/mL [5,10]. A stainless steel analytical symmetry column (ACE5C18 V17-1586, Avantor^®^, Lutterworth, UK) with 5 μm particle size (4.6 mm internal diameter × 250 mm length) packed with a dimethyl octylsilyl (C18)-bonded amorphous silica stationary phase was used. The mobile phase was a binary mixture of HPLC-grade acetonitrile and purified water at gradient ratio of 65:35–75:25% (*v*/*v*) that was blended within 26 min, freshly prepared for each run, and degassed before use. The injection volume was 10 μL, and the flow rate was 1 mL/min. The samples were estimated by ultraviolet detection at a wavelength of 250 nm. The EE percentage was calculated by Equation (1):

(1) $$\mathrm{EE}\%\;=\frac{\mathrm{zerumbone}\;\mathrm{in}\;\mathrm{extract}\;\mathrm{added}\;\mathrm{to}\;\mathrm{the}\;\mathrm{formulation}\;-\;\mathrm{zerumbone}\;\mathrm{amount}\;\mathrm{in}\;\mathrm{supernatant}}{\mathrm{zerumbone}\;\mathrm{in}\;\mathrm{extract}\;\mathrm{added}\;\mathrm{to}\;\mathrm{the}\;\mathrm{formulation}}\times 100.\;$$

#### 2.2.5. Antifungal Activities of Ethosomes Loaded with *Z. zerumbet* (L.) Rhizome Extract

The antifungal activities of the ethosomes loaded with different concentrations of *Z. zerumbet* (L.) rhizome extract were examined. The loaded *Z. zerumbet* (L.) rhizome extract was varied at 0.1, 0.25, 0.5, 1, 1.5, 2.5, and 5X the minimum fungicidal concentration (MFC; 62.5 µg/mL) of *Z. zerumbet* (L.) rhizome hexane extract, which was discovered in our previous study [5]. Broth dilution was used to test the sensitivity of *Candida albicans* for the ethosomes loaded with different concentrations of *Z. zerumbet* (L.) rhizome extract. For this process, Sabouraud dextrose broth (SDB) was prepared and diluted with purified water to achieve a turbidity level equivalent to the 0.5 McFarland standard at about 1 × 10^8^ CFU/mL *C. albicans*. The prepared SDB was then mixed with the ethosomes loaded with different concentrations of *Z. zerumbet* (L.) rhizome extract (6.25–312.5 µg/mL) in test tubes and kept in a 37 °C incubator for 24 h. The turbidity of the samples was determined and compared with that of the control. The lowest concentration of ethosome loaded with the extract that can inhibit microorganism growth was defined as the minimum inhibition concentration (MIC) [11].

The MFC of the ethosome loaded with extract was determined by streaking the turbid mixture from the MIC test onto the surface of Sabouraud dextrose agar (SDA), which was then incubated at 37 °C for 20–24 h. The lowest concentration of the ethosome loaded with extract that can inhibit the formation of fungal colonies on the SDA plate was defined as the MFC [11].

#### 2.2.6. In Vitro Skin Penetration Studies of Ethosomes Loaded with *Z. zerumbet* (L.) Rhizome Extract

In vitro skin penetration studies were conducted using vertical Franz diffusion cells. Neonatal porcine skin from piglets that died of natural causes and provided by a local pig farm in Chonburi province, Thailand, was used as the barrier membrane. Subcutaneous fat was removed using surgical blades and scissors. Prior to the experiment, the skin was washed with phosphate-buffered saline to remove any contaminants. Then the skin was cut to the Franz diffusion cell mount size, wrapped by aluminum foil, and stored at −10 °C before further experimentation. In order to set up the experiment, piglet skin was mounted between the donor and receptor chambers of diffusion cells. The stratum corneum was turned to face the donor side [12]. Phosphate buffer (pH 7.4)–ethanol (80:20) was applied as the medium. The in vitro experiment was run for 24 h at controlled temperature of 32 °C with agitation by a magnetic stirrer. The *Z. zerumbet* (L.) rhizome liquid extract (62.5 and 312.5 µg) or the ethosomes loaded with the extract (62.5 and 312.5 µg) was poured at the donor sides and covered by Parafilm. The diffused substance was sampled at 1 mL from the receptor side at 15, 30 min, 1 2, 3, 4, 6, 12, and 24 h. The zerumbone from the sample was analyzed by HPLC as mentioned in the EE method.

#### 2.2.7. In Vitro Skin Retention Studies of Ethosomes Loaded with *Z. zerumbet* (L.) Rhizome Extract

Following the in vitro skin penetration test, the used pig skin (n = 3) [13,14,15] was washed three times with deionized water for 15 s each and then wiped with a paper tissue. The stratum corneum layers were removed from the treated skin using the tape strip method [12]. The stratum corneum was peeled 30 times using a 24 mm wide pressure-sensitive adhesive tape (Scotch^®^ Transparent Tape 500, 3M Co., Ltd., Bangkok, Thailand). All of the stripped tapes were placed in glass vials containing 5 mL of ethanol and sonicated for 15 min. Afterward, 1 mL of the ethanol solution was drawn and centrifuged at 10,000 rpm at 25 °C for 30 min. Zerumbone was quantified by HPLC.

After the stratum corneum was removed, the remaining skin was sliced into small fragments and placed in a glass vial containing 3 mL of ethanol for 24 h. In brief, 1 mL of the collected ethanol was pipetted into a centrifuge tube and centrifuged at 10,000 rpm and 25 °C for 15 min. The zerumbone concentration in the resulting supernatant was determined using HPLC. The quantity of zerumbone in the viable epidermis and dermis was measured using Equation (2):

(2) $$\mathrm{Drug}\;\mathrm{amount}\;\mathrm{in}\;\mathrm{the}\;\mathrm{viable}\;\mathrm{epidermis}\;\mathrm{and}\;\mathrm{dermis}\;\left(µg/{\mathrm{cm}}^{2}\right)=\frac{R_{v}}{S}$$

where *R_v_* is the amount of zerumbone in the viable epidermis and dermis (µg), and *S* is the skin penetration area (cm^2^).

The enhancement ratio (*ER*) was calculated using Equation (3):

(3) $$ER=\frac{\mathrm{Drug}\;\mathrm{amount}\;\mathrm{in}\;\mathrm{the}\;\mathrm{viable}\;\mathrm{epidermis}\;\mathrm{and}\;\mathrm{dermis}\;\mathrm{of}\;\mathrm{ethosomal}\;\mathrm{formulation}}{\mathrm{Drug}\;\mathrm{amount}\;\mathrm{in}\;\mathrm{the}\;\mathrm{viable}\;\mathrm{epidermis}\;\mathrm{and}\;\mathrm{dermis}\;\mathrm{of}\;\mathrm{extracts}\;}$$

#### 2.2.8. Statistical Analysis

Data were statistically analyzed using one-way ANOVA, followed by Tukey’s honest significant difference post hoc test for the data from three independent experiments.

## 3. Results and Discussion

### 3.1. Vesicle Size, Size Distribution, and Zeta Potential

**Figure 1:**
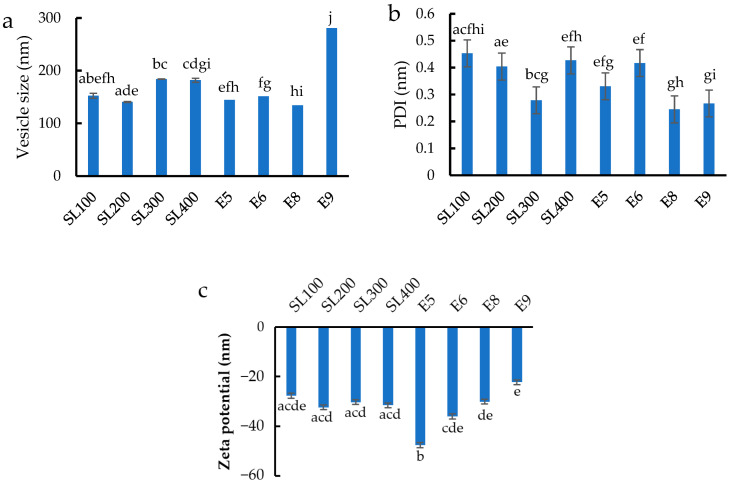
(**a**) Vesicle size, (**b**) size distribution, and (**c**) zeta potential of different formulations of blank ethosomes. Different letters above the error bars indicate significant differences at the 0.05 level (ANOVA and post hoc test).

The vesicle size, size distribution, and zeta potential of blank ethosomes are depicted in bar chart format and presented in Figure 1a–c, respectively. Most of the sizes ranged between 140.8 and 184.1 nm (Figure 1a), except for E9 formulation whose size was 280.9 nm. An excessive amount of ethanol in the ethosome structure may result in an unstable membrane because phospholipids dissolve rapidly in ethanol, causing the vesicles to enlarge significantly [16]. SL200 formulation produced the smallest vesicle size. The addition of 300 and 400 mg of soy lecithin considerably enlarged the vesicles, and this finding is consistent with previous research stating that an increasing phospholipid concentration slightly or moderately increases the vesicle size [17,18,19]. The recommended amount of phospholipids that should be included in an ethosomal formulation is between 0.5% and 5% [20], which was applied in our formulation. As shown in Figure 1b, all the polydispersity index (PDI) values were between 0.245 and 0.453 nm. The formulations of SL300, E8, and E9 yielded PDI values below 0.280, showing that the preparation and formulations in this study were able to generate particles with low size dispersion [21].

Vesicular charge is a crucial characteristic that can affect vesicular features, such as stability and vesicle–skin contact. Therefore, the zeta potential of the entire ethosome formulation was measured. The zeta potential of the blank ethosomes was negative and in the range of −31.57 to −27.73 mV (Figure 1c). This high negative charge could stabilize the nanoparticle/vesicle system [22]. The phosphate group in soy lecithin was responsible for the negative charge on the ethosome’s surface [23,24]. The zeta potential was unaffected by soy lecithin at a portion ranging from 100 mg to 400 mg. Finally, a high ethanol concentration in the formulation resulted in a low negative charge.

**Figure 2:**
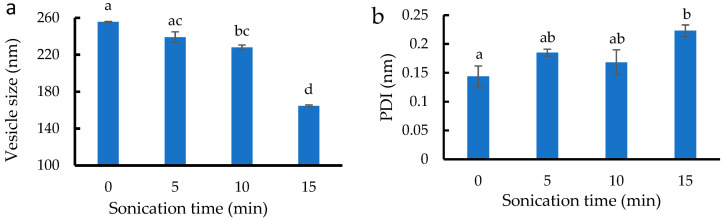

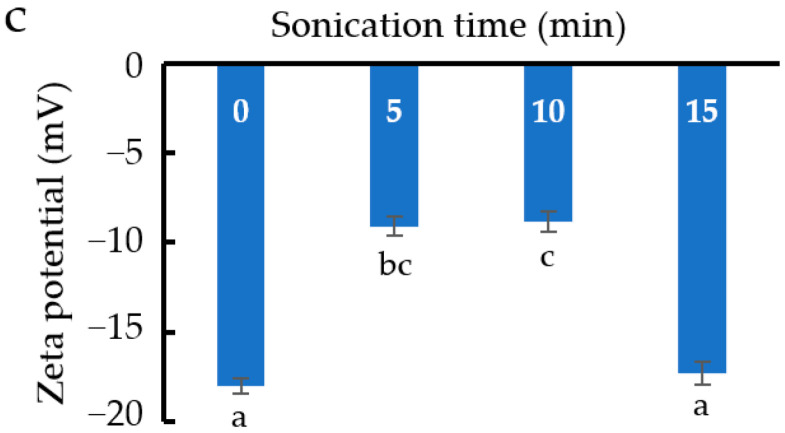
(**a**) Vesicle size, (**b**) size distribution, and (**c**) zeta potential of blank ethosomes treated by different sonication times. Different letters above the error bars indicate significant differences at the 0.05 level (ANOVA and post hoc test).

Owing to its small vesicle size, low PDI, and zeta potential lower than −30 mV, the ethosome with E8 formulation was chosen for further development through sonication. The effect of sonication period during preparation on the vesicle size, size distribution, and zeta potential of ethosomes is exhibited in Figure 2a–c, respectively. Sonication time reduced the vesicle size from 255.6 nm to 218.5 nm. At 15 min (5 min for three rounds), sonication slightly increased the PDI because this process breaks coarse drops into nanodroplets, hence producing ethosomes with a small, highly variable particle size [25]. The zeta potential altered with the length of time spent sonicating, which is consistent with the findings of another study regarding the effects of sonication on drug release, zeta potential, and pH [25].

**Figure 3:**
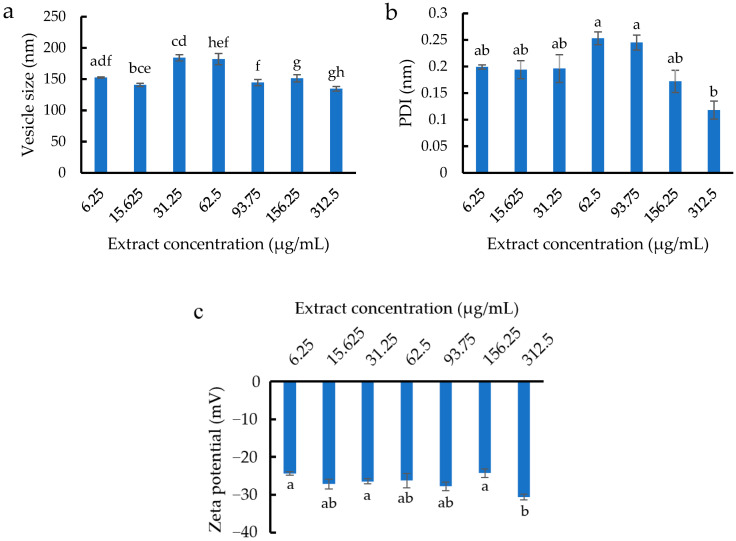
(**a**) Vesicle size, (**b**) size distribution, and (**c**) zeta potential of ethosomes loaded with different concentrations of *Z. zerumbet* (L.) rhizome extract. Different letters above the error bars indicate significant differences at the 0.05 level (ANOVA and post hoc test).

The E8 formulation with 15 min of sonication (5 min for three rounds) was chosen for loading with the active ingredient, *Z. zerumbet* (L.) rhizome extract (6.25–312.5 µg/mL). The vesicle size, size distribution, and zeta potential of ethosomes loaded with the extract are presented in Figure 3a–c, respectively. The size of ethosomes varied between 134.5 and 184.1 nm, and the PDI ranged from 0.118 to 0.253. The high extract concentration reduced the PDI, indicating that the loading of 6.25–312.5 µg/mL *Z. zerumbet* (L.) rhizome extract did not alter the physical properties of the ethosome.

### 3.2. Morphology

**Figure 4:**
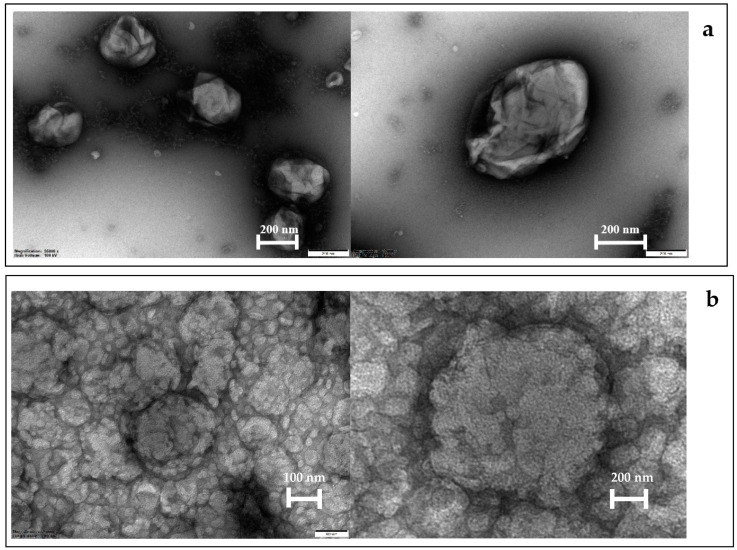
TEM of (**a**) blank ethosomes and (**b**) extract-loaded ethosomes.

The TEM images of the blank and extract-loaded ethosomes are shown in Figure 4a,b, respectively. The blank ethosomes had an irregular shape with an average diameter size of 200–400 nm. The vesicles shrunk and did not aggregate. Meanwhile, the morphology of extract-loaded ethosomes was spherical with gnarled surface. The vesicles were packed together, and their average size was around 150–400 nm. The TEM results for vesicle size were comparable with those from the vesicle size analyzer (Zetasizer), which measures particle size using dynamic light scattering. These results demonstrated that the ethosomes were generated using the cold procedure and subsequently filled with the *Z. zerumbet* (L.) rhizome extract.

### 3.3. Entrapment Efficiency of Ethosomes

The zerumbone in *Z. zerumbet* (L.) rhizome extract was quantified by HPLC and then monitored to evaluate the EE of the ethosomes as presented in Table 2. The herbal extract was added at two concentrations (156.25 and 312.5 µg/mL) to the E8 formulation. The EE percentage ranged from 24.42% to 31.58%, indicating that the developed ethosome was incapable of completely entrapping zerumbone. One possible reason is the high solubility of the active ingredient (zerumbone) in ethanol [26], leading to its dissolution in the solvent during the preparing of the ethosome. Moreover, the sonication process can significantly reduce the size of ethosomes. However, it might also affect the entrapment efficiency of the system [27]. Furthermore, some zerumbone may leak during centrifugation in the EE determination process [28]. These reasons can make the EE% slightly lower than in the previous study.

### 3.4. Antifungal Activities of Ethosomes Loaded with Z. zerumbet (L.) Rhizome Extract

**Figure 5:**
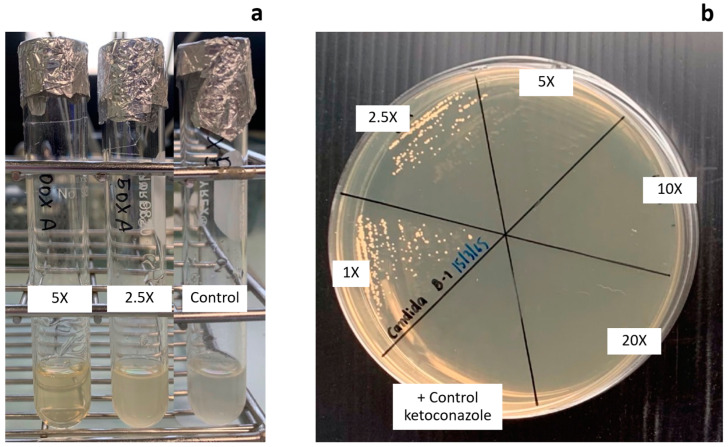
Antifungal test result of the ethosome loaded with different concentration of *Z. zerumbet* (L.) rhizome extract from (**a**) broth dilution technique test and (**b**) streaking onto the surface of Sabouraud dextrose agar.

Broth dilution was applied to determine the MIC of ethosomes loaded with *Z. zerumbet* (L.) rhizome extract against *C. albicans* (Figure 5a). The tested concentrations of *Z. zerumbet* (L.) rhizome extract loaded onto ethosome were between 6.25 and 312.5 µg/mL. The MIC of ethosomes loaded with *Z. zerumbet* (L.) rhizome extract was 312.5 µg/mL, which is five times higher than that of *Z. zerumbet* (L.) rhizome extract as reported previously [5]. As shown in Figure 5b, all incubated dilutions were then streaked on SDA plates to detect MFC. The MFC was discovered to be similar to the MIC, which has a concentration of 312.5 µg/mL, or five times that of the MFC hexane liquid extract. Compared with that in the liquid extract, the ability of *Z. zerumbet* (L.) rhizome extract in the ethosome to inhibit *C. albicans* growth was lower because a portion of the extract was not entrapped during ethosome preparation. Moreover, some active compounds entrapped inside the ethosome could retard the release or dissolution of the active ingredient to inhibit the growth of *C. albicans* [29].

### 3.5. In Vitro Skin Penetration Studies of Ethosomes Loaded with Z. zerumbet (L.) Rhizome Extract

**Figure 6:**
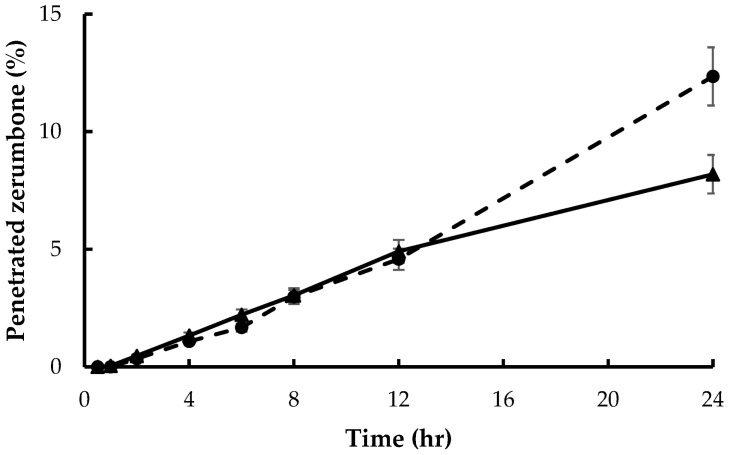
Time-integrated skin penetration percentage of zerumbone from the liquid extract (solid line) and the ethosomes loaded with *Z. zerumbet* (L.) rhizome extract (dash line) at 312.5 µg/mL.

Figure 6 illustrates the skin penetration ability of zerumbone from the liquid extract and the ethosome containing *Z. zerumbet* (L.) rhizome extract at a concentration of 312.5 µg/mL. During the initial 12 h, the penetration of zerumbone from both dosage forms was around 5%. After 24 h, the ethosomes significantly increased zerumbone’s penetration compared with the liquid extract.

### 3.6. In Vitro Skin Retention Studies of Ethosomes Loaded with Z. zerumbet (L.) Rhizome Extract

**Figure 7:**
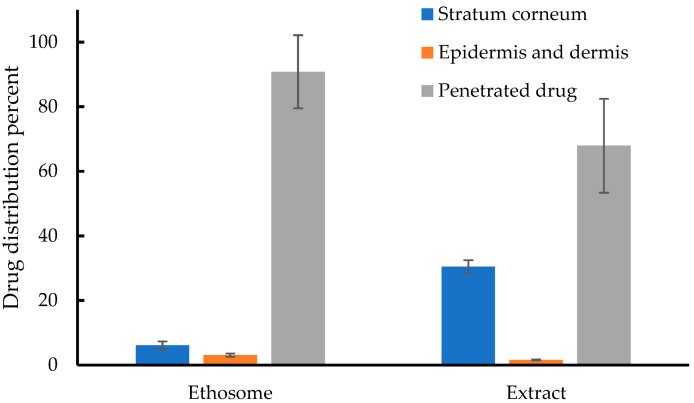
Retained ratio of zerumbone from the in vitro skin retention investigation in various skin layers.

The retention ratio of zerumbone in the stratum corneum, epidermis, dermis, and deeper layers of the skin was measured from the adhesive tape, small fragments of skin, and the medium (phosphate buffer) from the receptor chamber. After 24 h, the majority of zerumbone from the liquid extract and ethosome had penetrated through the porcine skin as illustrated in Figure 7. Owing to its chemical structure with low polarity [30], zerumbone can penetrate the medium after 24 h of testing. However, 30.48% of zerumbone was still present in the stratum corneum treated with the liquid extract. Meanwhile, the stratum corneum treated with the ethosome contained only 6.08% of zerumbone. This finding suggested that the ethosome can greatly increase zerumbone’s skin penetration into the deep layer skin of porcine models by interacting with the stratum corneum and disrupting its structure. According to one study, ethosomes with an average diameter of more than 600 nm were unable to reach the deep skin layers and stayed predominantly on the stratum corneum’s surface, and those with an average diameter of 300 nm were able to go further into the skin layers [31]. This finding was in agreement with the ethosome size in the current work at 134.5–184.1 nm, which is lower than 300 nm. Finally, the ER of the ethosome was 1.51, indicating that the zerumbone content in the viable epidermis and dermis was higher after the treatment of ethosome than after the treatment with the liquid extract. This finding confirmed the permeation-enhancing ability of the ethosome.

## 4. Conclusions

In this study, *Z. zerumbet* (L.) rhizome extract was successfully loaded onto ethosome systems. TEM and light scattering analysis showed that the ethosome size was less than 200 nm, making it an excellent candidate as permeation enhancer for drugs or active ingredients. Size distribution and zeta potential analysis suggested that the ethosome production method and formulation were stable and consistent. In addition, the ethosome loaded with *Z. zerumbet* (L.) rhizome extract prevented the growth of *C. albicans* and considerably improved the skin penetration and retention of zerumbone, the major compound of *Z. zerumbet* (L.) rhizome extract. This finding demonstrates the potential utilization of the ethosome loaded with *Z. zerumbet* (L.) rhizome extract in the treatment of deep skin fungal infections.

## Figures and Tables

**Table 1:** Formulation of blank ethosome.

Ingredients	Formulations							
	SL100	SL200	SL300	SL400	E5	E6	E8	E9
Soya lecithin (mg)	100	200	300	400	200	200	200	200
Ethanol (mL)	7	7	7	7	5	6	8	9
Polyethylene glycol 4000 (mg)	100	100	100	100	100	100	100	100
Water qs (mL)	20	20	20	20	20	20	20	20

**Table 2:** Entrapment efficiency of the ethosomes loaded with *Z. zerumbet* (L.) rhizome extract.

*Z. zerumbet* (L.) Rhizome Extract Concentration (µg/mL)	AUC	%Zerumbone Entrapment
156.25	1,375,327	24.42 ± 0.04
312.5	2,435,877	31.58 ± 0.05

## Data Availability

Not applicable.
